# Application of an Exploratory DBT–Based Psychotherapy Program for Suicide‐Related Symptoms in Chinese Outpatients With Affective Disorders

**DOI:** 10.1002/brb3.71827

**Published:** 2026-09-30

**Authors:** Chanchan Hu, Jingkai Chen, Zhong Wang, Xiaoyi Zhou, Yujin Yang, Shuguang Li, Ruili Zhang, Jianbo Lai, Jianbo Hu, Manli Huang, Shaohua Hu

**Affiliations:** ^1^ The First Affiliated Hospital Zhejiang University School of Medicine Hangzhou China; ^2^ The Key Laboratory of Mental Disorder's Management of Zhejiang Province Hangzhou China; ^3^ Department of Radiology, The First Affiliated Hospital, The Key Laboratory of Mental Disorder's Management of Zhejiang Province Zhejiang University School of Medicine Hangzhou China; ^4^ Oberlin College and Conservatory Oberlin Ohio USA

**Keywords:** affective disorders, DBT, psychotherapy, suicide

## Abstract

**Objective:**

This exploratory pilot study aimed to examine the potential effects of a shortened, individually delivered dialectical behavior therapy (DBT)‐based intervention added to pharmacotherapy on suicide‐related outcomes among Chinese outpatients with affective disorders.

**Methods:**

Twenty‐one participants recruited from the psychiatric outpatient clinics of the First Affiliated Hospital, School of Medicine, Zhejiang University, were randomly assigned to two groups. Participants in the research group received pharmacotherapy plus a shortened, individually delivered DBT‐based intervention, whereas those in the control group received pharmacotherapy alone. Participants completed baseline assessments, 12 weeks of treatment, and 12 weeks of follow‐up, with assessments performed every 4 weeks. Outcomes included suicide risk or severity, depression, anxiety, and hopelessness assessed using self‐report scales.

**Results:**

After adjustment for baseline values using repeated‐measures analysis of covariance (RM‐ANCOVA), the between‐group effect on suicide risk showed a marginal trend favoring the DBT‐based psychotherapy group, but did not reach conventional statistical significance (*F* = 4.26, *p* = 0.054). The time effect (*F* = 0.234, *p* = 0.965) and time‐by‐group interaction (*F* = 1.306, *p* = 0.261) for suicide risk were not statistically significant. Depressive symptoms showed a significant time effect across the follow‐up period, whereas most suicide‐related and hopelessness outcomes were not statistically significant. These findings should be interpreted as preliminary because of the small sample size and baseline imbalance.

**Conclusion:**

The results provided preliminary pilot evidence that a shortened DBT‐based intervention may be feasible and potentially beneficial as an adjunct to pharmacotherapy in this clinical population. However, the primary suicide‐risk outcome did not show a statistically significant between‐group effect. Larger adequately powered trials with baseline‐adjusted analyses and more comprehensive outcome assessment are needed before firm conclusions can be drawn.

## Graphical abstract

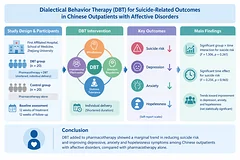

## 1 Introduction

Suicide occurs across lifespans and nationalities, with more than 700,000 people dying by suicide every year. As the fourth leading cause of death among 15‐ to 19‐year‐olds worldwide (World Health Organization 2026), suicide represents a severe public health problem. For each suicide death, more individuals engage in self‐harming behaviors, leaving tragic and long‐lasting consequences. The World Health Organization has recognized suicide as a public health priority and published a report named “Preventing suicide: A global imperative” to increase public awareness of the significance of suicide prevention (World Health Organization 2014). Several factors contribute to suicide risk, but psychiatric disorders are considered one of the most significant factors. In China, the prevalence and social burden of mental health disorders have increased significantly. Huang et al. (2019) surveyed 32,552 respondents from July 22, 2013, to March 5, 2015, and reported that the lifetime prevalence of any mental disorder in the general Chinese population was 16.6%; the 12‐month prevalence was 9.3%. Suicide risk is also substantial among individuals with mental disorders and may be particularly elevated in rural areas, with some reports estimating rates as high as 48%. The increased risk of suicidal behaviors among patients with mental disorders has therefore become an important social and public health concern.

Many mental disorders are strongly associated with suicidal ideation or behavior. Depression, for example, is a common mood disorder characterized by persistent sadness and loss of interest, and it is closely linked to suicide risk (Huang et al. 2019). According to the World Health Organization, more than 300 million people worldwide suffer from depression (Halverson et al. 2024). Depression is present in more than half of all suicide attempts, and the lifetime risk of suicide among patients with untreated depressive disorder has been reported to be nearly 20% (Halverson et al. 2024). Bipolar disorder is another affective disorder associated with elevated suicide risk. It is characterized by unusual shifts in mood, energy, activity levels, concentration, and functional capacity (National Institute of Mental Health). It is estimated that nearly 30% of individuals diagnosed with bipolar disorder attempt suicide at least once, and the suicide rate among bipolar patients is approximately 20 times that of the general population (Baldessarini et al. 2020).

Recent studies have documented both the high prevalence and complex risk profiles of suicidal and non‐suicidal self‐injurious behavior across diverse populations, underscoring the growing public health concern over self‐destructive behavior. For example, research from Pakistan revealed that deliberate self‐harm was strongly correlated with increased suicidal ideation and psychological distress, including symptoms of depression and anxiety, in both clinical and non‐clinical settings (Aqeel et al. 2021). These findings highlight the interplay between psychopathology and self‐harm tendencies (Aqeel et al. 2021). In addition to individual psychological factors, sociocultural and contextual stressors, such as the COVID‐19 pandemic, economic hardship, and social isolation, have also been associated with increased suicidal ideation among young people, suggesting that broader environmental influences may intensify vulnerability (Shoib et al. 2022).

Dialectical behavior therapy (DBT) is an evidence‐based psychotherapy that has been used to reduce suicidal and non‐suicidal self‐injurious behavior, particularly in individuals with recurrent suicide attempts and repeated self‐harm. The practice of DBT originated from the work of psychologist Marsha Linehan, and was influenced by Bandura's applied behaviorism principles and social learning theory (Turecki and Brent 2016). Initially developed for borderline personality disorder (BPD), DBT focuses on emotion regulation, interpersonal effectiveness, distress tolerance, mindfulness, and the development of more adaptive behavioral responses. As a suicide prevention approach, DBT emphasizes the identification of problems that contribute to suicidal and self‐injurious behavior, and supports patients in developing more effective coping and problem‐solving strategies. DBT is also grounded in the concept of building a “life worth living,” and incorporates several innovative features, including acceptance of transformation and technology, helping patients engage flexibly with emotional experiences, openly addressing patients’ suicide risk, and maintaining a validating therapeutic stance toward patient progress (Read 2015).

The efficacy of DBT for adolescents at high risk of suicide was examined by McCauley et al. (2018). In a randomized clinical trial involving 173 adolescents, DBT showed significant advantages over individual and group supportive therapy in reducing repeat suicide attempts, non‐suicidal self‐injury (NSSI), and self‐harm after treatment. Building on this literature, the present study aimed to examine the potential clinical utility of a shortened DBT‐based intervention for suicide‐related symptoms among Chinese patients with affective disorders over a short‐term treatment and follow‐up period.

The existing literature covers a wide range of populations, including patients diagnosed with BPD, individuals at high risk of suicide, and those suffering from other types of mental health conditions. Previous studies suggest that DBT may be particularly useful for reducing combined suicide risk and self‐harm behavior. Some studies attempted to shorten the duration of DBT‐based interventions, while others delivered interventions electronically to increase accessibility (Wilks et al. 2018; Wright et al. 2018). These adapted formats have shown preliminary effectiveness in different settings (Wilks et al. 2018; Wright et al. 2018). In China, suicide prevention remains an important clinical and public priority, particularly among adolescents and young adults (Chunmei et al. 2017). Nevertheless, evidence on the implementation of DBT in Chinese clinical settings remains limited, highlighting the need for further research in this context. The structured and skills‐based nature of DBT may facilitate its broader clinical application, and elements such as mindfulness and acceptance may be compatible with aspects of Eastern culture. Therefore, future studies are needed to examine the feasibility, applicability, and potential effectiveness of DBT‐based interventions in Chinese clinical populations.

This exploratory pilot study aimed to compare pharmacotherapy plus a shortened, individually delivered DBT‐based intervention with pharmacotherapy alone in Chinese outpatients with affective disorders, focusing on suicide‐related outcomes over a 12‐week intervention and a subsequent 12‐week follow‐up.

## 2 Methods

### 2.1 Participants

The study was conducted at the First Affiliated Hospital, School of Medicine, Zhejiang University, from October 2018 to December 2019. A total of 21 participants were recruited and randomly assigned to two groups using a computer‐generated random number list prior to treatment initiation. Randomization was performed by a designated research coordinator within the study team who was not involved in treatment delivery or outcome assessments. This coordinator was also responsible for maintaining the original study records. Clinical evaluation by outpatient psychiatrists was conducted to confirm eligibility only and did not influence group allocation. A total of 11 participants in the research group received drug therapy combined with DBT‐based psychotherapy, and 10 participants in the control group received drug therapy only. Group allocation was independent of clinical judgment regarding treatment suitability.

Inclusion criteria included (1) participants who were over 14 years old and had received at least a middle‐school education; (2) participants who were willing to cooperate for at least 12 weeks, as the research group would receive DBT‐based therapy once a week; (3) participants who had an affective disorder diagnosis, primarily including major depressive disorder and bipolar disorder; (4) participants who actively sought treatment from October 2018 to December 2019.

The First Affiliated Hospital, School of Medicine, Zhejiang University approved the study. All participants provided written consent.

### 2.2 Study Design

Both groups of participants underwent a baseline assessment at the beginning of the study. Beck Depression Inventory‐II (BDI), Beck Scale for Suicide Ideation (BSI), Beck Anxiety Inventory (BAI), and Beck Hopelessness Scale (BHS) were used as assessment scales (Beck et al. 1974, 1988, 1996; Beck and Steer 1991). All outcome measures were self‐report instruments; therefore, the possibility of response bias was considered when interpreting the findings. Assessors were blinded to group allocation during the assessment phase and were unaware of whether participants had been assigned to the DBT‐based psychotherapy group or the control group. All participants received continuous pharmacotherapy as part of standard psychiatric care. Medication regimens were stable for at least one month before study enrolment and remained unchanged in medication class and dosage throughout the study and follow‐up period. For participants with unipolar depression, selective serotonin reuptake inhibitors (SSRIs) were used. For participants with bipolar depression, lithium carbonate or quetiapine was used. The same medication‐stability requirement was applied to both the groups, and no medication class or dosage changes were made during the study period, supporting the comparability of pharmacotherapy between the DBT‐based psychotherapy group and the control group.

Participants in the research group received DBT‐based psychotherapy once weekly for 12 weeks. The same assessment scales were used every four weeks to collect outcome data from both the groups. After the 12‐week treatment phase, participants entered an additional 12‐week follow‐up, with assessment conducted either in person or by telephone.

The research group received a shortened, individually delivered DBT‐based intervention rather than the full comprehensive DBT model. The program retained core DBT principles, including mindfulness practice, emotion regulation, behavioral analysis of suicidal/self‐injurious urges, crisis planning, skills practice, and therapist validation, but did not include all components of standard DBT, such as a full skills‐training group or 24‐h telephone coaching. The DBT‐based intervention was delivered by four therapists. The therapists had an average of 7.5 years of clinical practice experience. To support treatment adherence and consistency, monthly group supervision and case discussions were conducted throughout the study period. Supervision was provided by a fixed senior supervisor with more than 15 years of clinical experience. These supervision procedures were used to review clinical cases, discuss intervention strategies, and promote adherence to DBT‐consistent principles.

The specific DBT procedure was as follows.

### 2.3 DBT Procedure

The research group received DBT in a set format; the procedure was as follows:

Session 1:

The therapists established therapeutic relationships and set therapy goals. As part of suicide assessment, therapists assessed suicidal ideation, suicide plans, self‐injury behaviors, and self‐injury plans. Assessment of self‐injurious behavior during therapy was conducted as part of routine clinical risk assessment and treatment planning and was not included as a study outcome. Therapists also investigated risk factors, attempted to avoid risk factors, reached a non‐suicide agreement with participants, and recorded emergency contact information. Participants were asked to contact their therapist before attempting suicide, self‐harm, or whenever they had urgent suicidal thoughts.

Session 2–4:

The therapists established an in‐depth understanding of the participants’ life history, analyzed the influence of participants’ past difficulties on current dilemmas, and promoted participants’ self‐awareness by inviting participants to participate in the analysis process. Therapists educated participants about the concept of mindfulness, and informed participants about the involvement of mindfulness exercises during the treatment.

Session 5–10:

The participants learned coping skills and completed the corresponding worksheets under therapists’ instruction. Before each therapy session, participants had the opportunity to discuss the homework and practice a mindfulness exercise with therapists. After evaluating participants’ progress, therapists introduced new coping skills to the participants.

Session 11 and 12:

The therapists reviewed and summarized the consultation process, and prepared to end the counseling relationship at the last meeting.

### 2.4 Assessment Scales

BDI‐II: The BDI is a brief, self‐report inventory designed to measure the severity of depression symptoms. It consists of 21 items to assess the intensity of depression in patients and the general population. Each item contains four statements arranged in increasing severity about a particular symptom of depression (Beck et al. 1996).

BSI: BSI is an evaluation of suicidal thinking that helps identify individuals at risk. It also helps measure a broad spectrum of attitudes and behaviors clinicians routinely consider when assessing a participant's suicide risk (Beck and Steer 1991).

BHS: BHS measures negative attitudes toward the future. Responding to the 20 true or false items on the (BHS), participants can either endorse a pessimistic statement or deny an optimistic statement. Research supports a positive relationship between BHS scores and measures of depression and suicidal ideation. The BHS is a valid predictor of potential suicide and is helpful in measuring the three major aspects of hopelessness: attitudes toward the future, loss of motivation, and expectations for the future (Beck et al. 1974).

BAI: BAI is a brief, self‐report assessment measuring anxiety severity. With the BAI, participants respond to 21 items rated on a scale from 0 to 3. Each item is a description of subjective, somatic, or panic‐related symptoms of anxiety (Beck et al. 1988).

All enrolled participants underwent 24‐week scale assessments. Validated Chinese versions of the BDI‐II, BAI, BSI, and BHS were used in this study, as supported by previous psychometric validation studies (Li et al. 2011; Ma et al. 2020; Che et al. 2006; Lu et al. 2002).

### 2.5 Statistical Analysis

Baseline demographic and clinical characteristics were compared between the DBT‐based psychotherapy group and the control group using independent‐samples *t*‐tests or chi‐square tests, as appropriate. Because several baseline clinical outcomes differed between groups, the primary analyses were updated using repeated‐measures analysis of covariance (RM‐ANCOVA) to adjust for baseline values. In these models, group was included as the between‐subject factor, time was included as the within‐subject factor, and the corresponding baseline score was included as a covariate. The main effects of time and group, as well as the time‐by‐group interaction, were examined. A two‐sided *p* value of less than 0.05 was considered statistically significant.

## 3 Results

### 3.1 Subject Disposition

The study population consisted of 21 participants: 11 were randomly assigned to the research group, with an average age of 17.45 ± 3.64. The other 10 were placed in the control group, with an average age of 16.20 ± 1.81. The relatively young mean age of both groups indicates that the study sample predominantly represented an adolescent and young clinical population. Demographic characteristics were broadly comparable; however, several baseline clinical characteristics were imbalanced between groups. The baseline suicide severity score was 2.38 ± 0.90 in the control group and 1.55 ± 0.53 in the research group (*p* = 0.017), and the baseline suicide risk score was 54.30 ± 21.87 in the control group and 31.09 ± 27.15 in the research group (*p* = 0.045). Because these baseline differences complicate direct interpretation of post‐treatment between‐group comparisons, the main analyses were updated using RM‐ANCOVA adjusted for corresponding baseline values. The demographic and baseline clinical characteristics of the participants are presented in Table 1.

**TABLE 1:** Demographic and baseline clinical characteristics.

	DBT (*N* = 11)	Control (*N* = 10)	*t*/*χ* ^2^	*p*
**Age (years)**	17.45 ± 3.64	16.20 ± 1.81	0.982	0.338
**Gender (male/female)**	3/8	1/9	1.014	0.314
**Education (years)**	12.45 ± 1.97	11.30 ± 2.83	1.094	0.288
**Ethnicity (Han/others)**	11/0	10/0		
**Age of onset**	15.18 ± 2.64	15.30 ± 3.06	−0.095	0.925
**Beck depression inventory**	20.45 ± 13.66	29.00 ± 9.78	−1.633	0.119
**Beck anxiety inventory**	30.09 ± 12.48	40.60 ± 7.60	−2.300	0.033^*^
**Beck hopelessness scale**	11.09 ± 4.70	14.70 ± 2.79	−2.111	0.048^*^
**Attitude toward the future**	3.72 ± 1.79	4.50 ± 1.96	−0.944	0.357
**Loss of motivation**	4.09 ± 2.47	5.40 ± 1.78	−1.382	0.183
**Expectations for the future**	3.27 ± 1.56	3.80 ± 1.55	−0.777	0.446
**Beck suicide inventory**				
** Severity of suicide**	1.55 ± 0.53	2.38 ± 0.90	−2.619	0.017^*^
** Suicide risk**	31.09 ± 27.15	54.30 ± 21.87	−2.143	0.045^*^

^*^ *p* < 0.05.

### 3.2 Primary Outcome: Suicide Risk

At baseline, the control group showed a higher suicide risk score than the DBT‐based psychotherapy group. The suicide risk score decreased from 31.09 ± 27.15 at baseline to 14.91 ± 21.62 at week 24 in the DBT‐based psychotherapy group, and from 54.30 ± 21.87 to 41.30 ± 25.76 in the control group. However, after adjustment for baseline suicide risk using RM‐ANCOVA, the time effect was not statistically significant (*F* = 0.234, *p* = 0.965), and the time‐by‐group interaction was also not statistically significant (*F* = 1.306, *p* = 0.261). The group effect showed a marginal trend favoring the DBT‐based psychotherapy group, but did not reach conventional statistical significance (*F* = 4.26, *p* = 0.054). For suicide severity, RM‐ANCOVA also showed no significant time effect (*F* = 0.541, *p* = 0.776), group effect (*F* = 2.55, *p* = 0.128), or time‐by‐group interaction (*F* = 1.313, *p* = 0.258). These findings suggest a possible trend toward lower suicide risk in the DBT‐based psychotherapy group after baseline adjustment, but this result should be interpreted cautiously. The RM‐ANCOVA results for suicide severity and suicide risk are presented in Table 2.

**TABLE 2:** Beck suicide inventory.

Measures	DBT (*N* = 11)	Control (*N* = 10)	*p*	Time	Group	Time × Group
**Severity of suicide**						
** Baseline**	1.55 ± 0.53	2.38 ± 0.90	0.017^*^	*F* = 0.541	*F* = 2.55	*F* = 1.313
**Intervention period (Weeks 4–12)**						
** 4 weeks**	1.55 ± 0.49	2.30 ± 0.92	0.029^*^	*p* = 0.776	*p* = 0.128	*p* = 0.258
** 8 weeks**	1.49 ± 0.50	2.10 ± 1.05	0.102			
** 12 weeks**	1.47 ± 0.49	1.93 ± 1.02	0.199			
**Follow‐up period (Weeks 16–24)**						
** 16 weeks**	1.38 ± 0.50	1.87 ± 1.03	0.176			
** 20 weeks**	1.33 ± 0.48	1.81 ± 1.05	0.185			
** 24 weeks**	1.29 ± 0.48	1.79 ± 1.04	0.166			
**Suicide risk**						
** Baseline**	31.09 ± 27.15	54.30 ± 21.87	0.033^*^	*F* = 0.234	*F* = 4.26	*F* = 1.306
**Intervention period (Weeks 4–12)**						
** 4 weeks**	26.73 ± 23.20	53.90 ± 21.79	0.055	*p* = 0.965	*p* = 0.054	*p* = 0.261
** 8 weeks**	26.18 ± 22.21	50.10 ± 21.06	0.110			
** 12 weeks**	23.09 ± 18.92	47.00 ± 20.62	0.084			
**Follow‐up period (Weeks 16–24)**						
** 16 weeks**	19.27 ± 18.96	44.60 ± 21.67	0.111			
** 20 weeks**	13.55 ± 19.17	41.30 ± 25.76	0.125			
** 24 weeks**	14.91 ± 21.62	41.30 ± 25.76	0.128			

^*^ *p* < 0.05.

### 3.3 Secondary Outcomes: Treatment Effectiveness

After baseline adjustment, depressive symptoms showed a significant time effect across the follow‐up period (*F* = 18.221, *p* < 0.001), suggesting an overall reduction in depressive symptoms over time. However, neither the group effect (*F* = 3.270, *p* = 0.086) nor the time‐by‐group interaction (*F* = 0.676, *p* = 0.468) was statistically significant. For anxiety symptoms, no significant time effect (*F* = 0.041, *p* = 1.000), group effect (*F* = 3.22, *p* = 0.090), or time‐by‐group interaction (*F* = 1.883, *p* = 0.090) was observed after baseline adjustment. Similarly, the BHS total score did not show significant adjusted time, group, or time‐by‐group effects. Some BHS subdomains showed numerical improvement over time, but these changes should be interpreted cautiously because they were not consistently significant after adjustment for baseline values. The results for derpession and anxiety are presented in Table 3; the results for hopelessness and its subdomains are presented in Table 4.

**TABLE 3:** The treatment effect in two treatment groups.

Measures	DBT (*N* = 11)	Control (*N* = 10)	*p*	Time	Group	Time × Group
**Beck‐depression**						
** Baseline**	20.45 ± 13.67	29.00 ± 9.78	0.119	*F* = 18.221	*F* = 3.270	*F* = 0.676
**Intervention period (Weeks 4–12)**						
** 4 weeks**	20.18 ± 11.83	26.30 ± 9.09	0.203	*p* < 0.001	*p* = 0.086	*p* = 0.468
** 8 weeks**	17.82 ± 11.26	23.60 ± 8.99	0.212			
** 12 weeks**	15.00 ± 10.43	21.50 ± 9.22	0.148			
**Follow‐up period (Weeks 16–24)**						
** 16 weeks**	11.82 ± 11.11	20.40 ± 9.13	0.070			
** 20 weeks**	10.27 ± 11.28	19.70 ± 9.56	0.054			
** 24 weeks**	9.91 ± 11.30	19.20 ± 9.85	0.060			
**Beck‐anxiety**						
** Baseline**	30.09 ± 12.48	40.60 ± 7.60	0.033^*^	*F* = 0.041	*F* = 3.22	*F* = 1.883
**Intervention period (Weeks 4–12)**						
** 4 weeks**	30.36 ± 13.99	35.60 ± 5.34	0.055	*p* = 1.000	*p* = 0.090	*p* = 0.090
** 8 weeks**	29.00 ± 13.86	33.30 ± 5.87	0.110			
** 12 weeks**	28.09 ± 14.00	30.70 ± 6.58	0.084			
**Follow‐up period (Weeks 16–24)**						
** 16 weeks**	26.64 ± 14.35	29.50 ± 5.89	0.111			
** 20 weeks**	26.00 ± 14.28	28.10 ± 5.96	0.125			
** 24 weeks**	25.64 ± 14.39	27.60 ± 5.50	0.128			

^*^ *p* < 0.05.

**TABLE 4:** Beck hopeless scale.

Measures	DBT (*N* = 11)	Control (*N* = 10)	*p*	Time	Group	Time × Group
**Total score**						
** Baseline**	11.09 ± 4.70	14.70 ± 2.79	0.048^*^	*F* = 0.284	*F* = 0.068	*F* = 1.833
**Intervention period (Weeks 4–12)**						
** 4 weeks**	11.27 ± 4.78	14.40 ± 2.76	0.086	*p* = 0.943	*p* = 0.798	*p* = 0.099
** 8 weeks**	10.82 ± 4.81	13.50 ± 3.53	0.166			
** 12 weeks**	9.36 ± 5.08	11.20 ± 4.61	0.399			
**Follow‐up period (Weeks 16–24)**						
** 16 weeks**	7.36 ± 5.64	11.30 ± 5.01	0.109			
** 20 weeks**	6.09 ± 5.41	11.00 ± 5.51	0.054			
** 24 weeks**	5.54 ± 5.37	10.90 ± 5.65	0.038^*^			
**Attitude toward the future**						
** Baseline**	3.73 ± 1.79	4.50 ± 1.96	0.357	*F* = 11.979	*F* = 0.926	*F* = 0.485
**Intervention period (Weeks 4–12)**						
** 4 weeks**	3.82 ± 1.83	4.30 ± 2.00	0.572	*p* < 0.001	*p* = 0.348	*p* = 0.601
** 8 weeks**	3.64 ± 2.01	4.30 ± 2.00	0.459			
** 12 weeks**	3.18 ± 1.94	3.80 ± 1.75	0.455			
**Follow‐up period (Weeks 16–24)**						
** 16 weeks**	2.64 ± 2.16	3.70 ± 1.83	0.240			
** 20 weeks**	2.55 ± 2.11	3.50 ± 1.84	0.286			
** 24 weeks**	2.55 ± 2.01	3.40 ± 1.78	0.318			
**Loss of motivation**						
** Baseline**	4.09 ± 2.47	5.4 ± 1.78	0.183	*F* = 12.726	*F* = 1.905	*F* = 0.489
**Intervention period (Weeks 4–12)**						
** 4 weeks**	4.18 ± 2.48	5.10 ± 1.73	0.343	*p* < 0.001	*p* = 0.184	*p* = 0.585
** 8 weeks**	3.91 ± 2.43	4.70 ± 2.00	0.428			
** 12 weeks**	3.18 ± 2.40	4.20 ± 2.10	0.316			
**Follow‐up period (Weeks 16–24)**						
** 16 weeks**	2.55 ± 2.21	3.70 ± 2.36	0.261			
** 20 weeks**	2.18 ± 2.14	3.70 ± 2.36	0.138			
** 24 weeks**	1.91 ± 1.92	3.60 ± 2.46	0.094			
**Expectations for the future**						
** Baseline**	3.27 ± 1.56	3.80 ± 1.55	0.033^*^	*F* = 0.295	*F* = 0.066	*F* = 1.971
**Intervention period (Weeks 4–12)**						
** 4 weeks**	3.27 ± 1.56	4.00 ± 1.83	0.055	*p* = 0.938	*p* = 0.800	*p* = 0.076
** 8 weeks**	3.27 ± 1.56	3.50 ± 1.78	0.110			
** 12 weeks**	3.00 ± 1.55	2.80 ± 2.04	0.084			
**Follow‐up period (Weeks 16–24)**						
** 16 weeks**	2.27 ± 1.79	3.00 ± 1.89	0.111			
** 20 weeks**	1.82 ± 1.78	2.70 ± 2.11	0.125			
** 24 weeks**	1.64 ± 1.86	2.80 ± 2.04	0.128			

^*^ *p* < 0.05.

Overall, the adjusted results indicate that depressive symptoms improved over time across the study period, whereas the adjusted between‐group effects for suicide risk, suicide severity, anxiety, and hopelessness were not statistically significant. The DBT‐based psychotherapy group showed a marginal trend toward lower suicide risk compared with pharmacotherapy alone, but this trend did not meet conventional statistical significance after baseline adjustment. Therefore, the results should be interpreted as exploratory pilot findings rather than confirmatory evidence of treatment efficacy.

## 4 Discussion

Bipolar disorder is associated with one of the highest suicide risks among psychiatric disorders, with suicide rates estimated to be 20–30 times that of the general population. Approximately one‐third to one‐half of individuals with bipolar disorder attempted suicide at least once in their lifetime. Among individuals with depression, suicide rates are estimated at 4%–10.6%, and a lifetime suicide risk of approximately 6% (Hawton and van Heeringen 2009). DBT is an evidence‐based psychotherapy technique that has been shown to reduce suicide‐related risk and may also benefit patients with refractory depression, eating disorders, alcohol addiction, drug addiction, and emotional disorders.

This exploratory pilot study randomly assigned participants into the pharmacotherapy‐only group and the pharmacotherapy plus DBT‐based psychotherapy group. In the descriptive results, participants in the DBT‐based psychotherapy group showed numerical reductions in suicide risk and suicide severity during the 24‐week observation period. However, after using RM‐ANCOVA to adjust for baseline differences between groups, the previously observed significant between‐group effect on suicide risk was attenuated and no longer reached conventional statistical significance. The adjusted group effect for suicide risk showed only a marginal trend favoring the DBT‐based psychotherapy group. Therefore, the present findings should be interpreted as a preliminary evidence, suggesting potential clinical benefit rather than confirmatory evidence of treatment efficacy.

DBT, an outstanding treatment technique in the third wave of cognitive‐behavioral therapy development, has been studied across various psychiatric populations. Its clinical application is not limited to suicide risk reduction. For example, Lynch et al. (2003, 2015) reported that DBT was associated with reduced depressive symptoms and improvements in personality disorder symptoms. Wright et al. (2018) also conducted a randomized controlled study of adapted DBT for bipolar disorder and found beneficial effects on mood symptoms. Previous studies suggest that DBT may produce sustained benefits beyond the acute treatment phase, particularly in reducing recurrent suicidal behaviors and improving emotion regulation (Mehlum et al. 2019). Follow‐up studies have shown that gains achieved through DBT can be maintained for several months to years, especially when core skills are internalized and practiced consistently (Calati and Courtet 2016; Mehlum et al. 2016).

The practice of DBT has demonstrated meaningful improvement in treating relevant disorders. Thus, in clinical psychology, the implementation of DBT‐based psychotherapy for people with suicide‐related symptoms is meaningful.

This study provides a preliminary contribution to the existing body of knowledge by extending evidence for the effectiveness of DBT beyond Western populations. The numerical trends observed in the DBT‐based psychotherapy group suggest that a time‐limited and adapted DBT‐based intervention may have potential clinical value in settings where long‐term psychotherapy resources are constrained. Despite the exploratory nature of this study, several strengths should be noted. First, this study addresses a clinically important, but relatively under‐studied topic by examining a DBT‐based intervention for suicide‐related symptoms in Chinese patients with affective disorders. Second, the study was conducted in a real‐world outpatient psychiatric setting, which increases its clinical relevance and reflects practical constraints commonly encountered in routine mental health care. Third, the 24‐week observation period, including both the 12‐week intervention phase and the 12‐week follow‐up phase, allowed us to explore symptom changes beyond the active treatment period. In addition, all participants received pharmacotherapy, which reflects common clinical practice and allowed us to examine whether a DBT‐based intervention may provide additional value as a supplemental psychosocial treatment. Finally, the use of baseline‐adjusted RM‐ANCOVA provided a more conservative and methodologically appropriate analysis to account for baseline imbalance between groups.

However, several potential limitations remain to be considered. First, all outcome measures in this study were assessed using self‐report scales, which may be subject to recall bias, social desirability bias, and limited insight (Zimmerman 2024), potentially affecting the accuracy of symptom severity and suicide risk assessment. Second, the relatively small sample size limits statistical power and may not adequately represent the broader population of patients with affective disorders in China. Because only 21 participants were included, the observed effects may be unstable and should be interpreted as preliminary pilot evidence rather than definitive efficacy evidence. Moreover, due to the limited sample size, randomization resulted in uneven distributions of demographic and baseline clinical characteristics, which may have further restricted the generalizability. Another limitation concerns the uneven distribution of baseline suicide‐risk scores. Several participants had a baseline suicide‐risk score of zero, whereas others had markedly elevated scores. This distribution may have affected the baseline mean values, increased within‐group variability, and contributed to floor effects among participants with no measurable suicide risk at baseline. As a result, some participants had limited room for further improvement, while others had greater potential for score reduction. These factors may have reduced the stability of the observed treatment effects and partly explain why some outcome changes were less robust or did not reach statistical significance. Fourth, depressive disorder and bipolar disorder may differ substantially in suicide risk, symptom development, and response to DBT; therefore, analyzing them as a combined group represents an additional limitation of this study. Also, there were significant differences in participants’ symptoms at baseline due to the limited sample size. The participants in the control group initially had milder symptoms than the research group, which may also influence the interpretation of our data. Fifth, the age distribution of the sample should also be considered when interpreting the findings. Most participants were adolescents or young individuals, and the mean age was below 18 years in both groups. Developmental differences in emotion regulation, suicidality, treatment engagement, and response to psychotherapy may limit the generalizability of these findings to adult populations with affective disorders. Future studies should examine adolescent and adult populations separately and include larger age‐stratified samples. Finally, we implemented a 12‐week DBT‐based intervention, which is substantially shorter than conventional comprehensive DBT programs that usually last 6–12 months. Therefore, the intervention should be described as a shortened, individually delivered DBT‐based psychotherapy program rather than standard comprehensive DBT. We adopted the adjusted format to better accommodate local customs. Since psychotherapy in China is not covered by governmental medical care, many patients and their families find long‐term treatment enormously challenging. In addition, this study did not consider whether participants had a comorbidity personality disorder, which might also negatively affect the data analysis. All the above factors may influence the external validity of the results, and we hope to replicate and improve the study design in future work. Based on the current results, we look forward to furthering our study by expanding the participant pool, expanding the intervention scale, identifying the influential factors in DBT for suicide risk, and evaluating the long‐term effects of DBT‐based intervention.

## 5 Conclusion

This exploratory pilot study suggests that a shortened DBT‐based psychotherapy intervention may be feasible and may have potential clinical value for Chinese patients with affective disorders when delivered as an adjunct to pharmacotherapy. However, after adjustment for baseline differences using RM‐ANCOVA, the between‐group effects on suicide risk and suicide severity were no longer statistically significant, although a marginal trend favoring the DBT‐based psychotherapy group was observed for suicide risk. Given the small sample size, baseline imbalances, reliance on self‐report scales, and limited pharmacotherapy detail, the findings should be interpreted cautiously. Larger, adequately powered randomized trials with stratified randomization based on suicide‐risk severity, clearer medication monitoring, and more comprehensive outcome assessment are needed to confirm the efficacy and generalizability of DBT‐based interventions in this population.

## Author Contributions

**Shaohua Hu**, **Manli Huang**, and **Jianbo Hu** contributed to conceptualization and supervision. **Zhong Wang** and **Xiaoyi Zhou** contributed to data curation and psychotherapy methodology. **Jianbo Lai** contributed to data curation, writing, review, and editing. **Yujin Yang** contributed to the original draft preparation. **Shuguang Li** contributed to formal analysis and data curation. **Jingkai Chen** contributed to formal analysis. **Chanchan Hu** contributed to conceptualization, writing, review and editing, psychotherapy methodology, and funding acquisition. **Ruili Zhang** contributed to data curation.

## Funding

This work was supported by Medical Technology and Education of Zhejiang Province of China under Grant LGC22H090001, with Chanchan Hu as the principal investigator. The total project budget was CNY 50,000, with no joint funding or self‐raised funds. The funder had no role in the study design, data collection, data analysis and interpretation, manuscript preparation, or the decision to submit the article for publication.

## Ethics Statement

This study has received approval from the Clinical Research Ethics Committee of the First Affiliated Hospital, Zhejiang University School of Medicine (Reference number: IIT20200779A).

## Conflicts of Interest

The authors declare no conflicts of interest.

## Supporting information




**Supplementary Material**: brb371827‐sup‐0001‐SuppMat.docx

**Supplementary Material**: brb371827‐sup‐0002‐SuppMat.jpg

**Supplementary Material**: brb371827‐sup‐0003‐SuppMat.pdf

## Data Availability

The data that support the findings of this study are available in the Supporting Information of this article.
