# Supplementary material for: Application of an Exploratory DBT–Based Psychotherapy Program for Suicide‐Related Symptoms in Chinese Outpatients With Affective Disorders
Source: Brain Behav. 2026 Sep 30;16(10):e71827. doi: 10.1002/brb3.71827 (PMC13627894; doi:10.1002/brb3.71827)
Supplement: Supplementary file 1 [file BRB3-16-e71827-s001.pdf]

LGC22H090001

浙江省基础公益研究计划

项目计划书

**立项编号：**	LGC22H090001
**项目名称：**	基于近红外脑功能成像系统（fNIRS）的抑郁症心理治疗疗效生物学机制研究
**专项类别：**	公益项目
**项目类别：**	分析测试项目
**项目负责人：**	胡婵婵　　**电话：** 18868735043
**电子邮箱：**	violahu@126.com
**通信地址：**	庆春路79号
**邮政编码：**	325000
**依托单位：**	浙江大学
**申报日期：**	2021-12-10

浙江省科学技术厅

浙江省自然科学基金委员会办公室

2021 年制

审核通过

填写说明

一、请认真阅读省基础公益研究计划有关项目和经费管理办法，按要求认真填写《浙江省基础公益研究计划项目计划书》（简称《计划书》）。填写《计划书》时要求科学严谨、实事求是、表述清晰、准确，并认真阅读本填报说明。

二、项目负责人应当按照申请书的内容填写《计划书》，除根据确定的资助额度对项目经费预算进行适当调整外，不得对申请书的其他内容进行变更。依托单位应对《计划书》内容进行审核。

三、《计划书》经项目负责人和依托单位签字盖章，并经省自然科学基金委员会办公室审核批准后，将作为项目执行、检查、验收的依据。

四、资助项目的有关研究成果，包括论文、专著、专利、获奖等情况，均须按规定标注“浙江省基础公益研究计划项目”（属于省自然科学基金的应标注“浙江省自然科学基金项目”）和立项编号。

五、省基础公益研究计划的项目经费管理（包括省级财政拨款经费、联合资助经费、自筹经费）依照省财政关于科技项目的有关经费管理要求执行。

基本信息

负责人信息	姓名	胡婵婵	性别	女	出生日期	1990-09-05
	电话	18868735043	E-mail		violahu@126.com	
	证件类型	身份证18位	证件号码		330328199009055527	
项目基本信息	项目名称	基于近红外脑功能成像系统（fNIRS）的抑郁症心理治疗疗效生物学机制研究				
	英文名称	Research on the Biological Mechanism of the Efficacy of Psychotherapy for Depression Based on the Near-Infrared Functional Imaging System (fNIRS)				
	专项类别	公益项目	项目类别		分析测试项目	
	项目研究阶段	试验发展				
	国家自然科学基金学科代码	H0925				
	国家自然科学基金学科名称	医学科学部/神经系统和精神疾病/精神疾病的心理测量和评估				
	国家标准学科分类代码	1904520				
	国家标准学科分类名称	心理学/心理测量/心理测量技术				
	预计研究年限	2022-01-01 - 2024-12-31				
	项目总经费	5.00	其中省财政资助经费		5.00	
	中文关键词	近红外脑功能成像系统；抑郁症；生物学机制；心理治疗				
	英文关键字	Functional Near-Infrared Spectroscopy; fNIRS; Depression; Biological Mechanism; Psychotherapy				

审核通过

中文摘要	本研究将 fNIRS 作为心理治疗的生物学标志，我们提出如下科学假说：抑郁症患者不同脑区脑血流中氧合血红蛋白和脱氧血红蛋白水平随着疾病进展和治疗存在水平上的波动，不同脑区的激活水平也将随之改变。神经影像学在抑郁症大脑结构生物学机制的研究认为抑郁症在边缘系统、海马、前扣带回皮层、背外侧前额叶皮层和基底神经节等情绪处理区域存在灰质体积减少。以往的神经影像学研究发现在fMRI下成功的 CBT 应该伴随大脑内灰质体积的变化。人际关系治疗（IPT）是CBT 第三浪潮的组成部分，抑郁症的IPT治疗在基于认知任务的 fNIRS 应该会有更显著的表达。<br>在本项目中，我们将在抑郁症患者开展药物治疗、心理治疗、药物治疗合并心理治疗，其中心理治疗使用人际关系治疗技术；另一方面，我们将对所有患者进行 fNIRS 全程评估，用以评估患者的脑功能变化，明确心理治疗的疗效水平；此外，我们还将使用规范心理评估对患者进行评估，比对 fNIRS 的评估有效性。从而明确心理治疗在抑郁症患者的疗效，以及不同治疗方法的疗效差异，明确心理治疗的生物学标志。

项目组成员

编号	姓名	成员类别	证件号码	性别	职称	学位	单位名称	电话	项目分工
1	胡婵婵	会员	330328199009055527	女	中级/主管技师	学士	浙江大学	18868735043	负责人
2	丁凯景	会员	330782198709190063	女	中级/主治医师	硕士	杭州市第七人民医院	15868839870	样本入组
3	陈京凯	会员	330302199009222010	男	中级/主管技师	学士	浙江大学	18667045770	数据分析，心理治疗
4	王中	会员	341023198812216035	男	中级/主管技师	硕士	浙江大学	18357132002	心理治疗，仪器操作
5	胡健波	会员	33032419820618431X	男	副高级/副主任技师	硕士	浙江大学	13957162871	质量把控
6	来建波	会员	330108199103160511	男	助理级/医师	硕士	浙江大学	13732254858	样本入组，论文撰写
7	周笑一	会员	330206199011103424	女	中级/主管技师	硕士	浙江大学	15158866089	心理治疗，仪器操作

审核通过

项目经费

项目总经费5.00万元，其中省财政资助经费5.00万元（第一批财政拨款5.00万元，第二批财政拨款0.00万元），联合资助经费0.00万元，自筹经费0.00万元。

	费用名称	金额
直接经费	设备费	0
	材料费	0
	测试化验加工费	0
	燃料动力费	0
	会议/差旅/国际合作交流费	1
	出版/文献/信息传播/知识产权事务费	0.5
	劳务费	1.7
	专家咨询费	0.8
	其他支出	0
间接经费	间接经费	1
	合计	5.00

需增添的仪器及设备

无

审核通过

研究计划

2022

研究内容：完成项目所有准备工作并开展入组，包括心理治疗一致性培训，心理测评一致性培训，fNIRS 规范化操作。

研究目标：为抑郁症的心理治疗找到一个明确的生物学指标，明确不同治疗方法的疗效和作用。最终将有利于推动抑郁症诊疗模式发展，协助我们选择更有效的抑郁症治疗方法，最终促进减轻抑郁症导致的社会负担。

2023

研究内容：进行中期研究质量把关和数据的初步分析，召开中期专家会 1 次。参与全国性学术会议交流 1 次。继续完善入组和数据的跟踪分析。

研究目标：进行数据分析，比对不同治疗方法的疗效，并明确 fNIRS 的生物学标志，以及 fNIRS 生物学标志不同阶段的水平。最终将有利于推动抑郁症诊疗模式发展，协助我们选择更有效的抑郁症治疗方法，最终促进减轻抑郁症导致的社会负担。

2024

研究内容：进行数据分析，比对不同治疗方法的疗效，并明确 fNIRS 的生物学标志，以及 fNIRS 生物学标志在治疗不同阶段的水平。

研究目标：基于 fNIRS 评估心理治疗对抑郁症的疗效，有效补充了精神检查和量表评估，为心理治疗的疗效提供更明确的生物学指标。最终将有利于推动抑郁症诊疗模式发展，协助我们选择更有效的抑郁症治疗方法，最终促进减轻抑郁症导致的社会负担。

预期研究成果

（1）明确不同治疗方法的疗效差异，探索在生物学水平有效的心理治疗方法。为抑郁症的心理治疗疗效明确生物学水平的评估方法。

（2）发表 SCI 论文 1 篇。参与全国性学术交流 1 次。

（3）最终推动抑郁症诊疗模式发展，协助我们选择更有效的抑郁症综合诊疗方法，最终促进减轻抑郁症导致的社会负担，为抑郁症的心理治疗明确生物学水平的标志！

审核通过

研究年限期间预期完成的成果

<table>
<tr><td colspan="9">一、预期成果产出情况</td></tr>
<tr><td>论著</td><td>发表科技论文</td><td>1 篇</td><td>出版科技著作</td><td>0 部</td><td colspan="4">共计出版科技专著：0 万字</td></tr>
<tr><td>专利</td><td>发明专利申请</td><td>0 件</td><td>实用新型专利申请</td><td>0 件</td><td>发明专利授权</td><td>0 件</td><td>实用新型专利授权</td><td>0 件</td></tr>
<tr><td rowspan="2">技术标准</td><td>国际标准</td><td>0 项</td><td>国家标准</td><td>0 项</td><td>行业标准</td><td colspan="3">0 项</td></tr>
<tr><td>地方标准</td><td>0 项</td><td>企业标准</td><td colspan="5">0 项</td></tr>
<tr><td colspan="9">二、本课题预期人才培养情况</td></tr>
<tr><td colspan="4">研究期限内项目组成员晋升职称人数：0</td><td colspan="5">研究期限内参与本项目的毕业研究生人数：0</td></tr>
<tr><td colspan="9">三、预期成果转化情况</td></tr>
<tr><td colspan="9">是否能实现成果转化：能</td></tr>
<tr><td rowspan="3">成果转化形式</td><td>新产品</td><td>0 件</td><td>新工艺</td><td>0 项</td><td>新技术</td><td>1 项</td><td>新品种</td><td>0 项</td></tr>
<tr><td colspan="8">开创性的产品或技术：为抑郁症的心理治疗疗效明确生物学水平的评估技术，辅助临床诊疗工作</td></tr>
<tr><td colspan="8">替代国外进口的产品或技术： 无</td></tr>
<tr><td rowspan="2">经济效益</td><td colspan="2">提升销售</td><td colspan="6">0 万元，测算依据：无</td></tr>
<tr><td colspan="2">产生利税</td><td colspan="6">0 万元，测算依据：无</td></tr>
</table>

<table>
<tr><td rowspan="2">环境治理</td><td>节能</td><td>否</td><td>节水</td><td>否</td><td>减排废气</td><td>否</td></tr>
<tr><td>减排废物</td><td>否</td><td>减排废水</td><td colspan="3">否</td></tr>
<tr><td rowspan="2">治理能力</td><td colspan="4">对公共卫生起到明显提升作用：否</td><td colspan="2">对公共安全起到明显提升作用：是</td></tr>
<tr><td colspan="4">对社会治理起到明显提升作用：是</td><td colspan="2">对防灾减灾起到明显提升作用：否</td></tr>
</table>

审核通过

签字和盖章页

我接受浙江省基础公益研究计划的资助，将按照项目申报书、批准通知和计划书负责实施本项目，严格遵守浙江省基础公益研究计划相关项目和经费管理规定，切实保证研究工作时间，认真开展研究工作，按时报送有关材料，及时报告重大情况变动，对资助项目发表的论著和取得的研究成果按规定进行标注。

项目负责人（签字）：

2022年 1 月 11 日

我单位同意承担上述浙江省基础公益研究计划项目，将保证项目负责人及其研究队伍的稳定和研究项目实施所需的条件，将严格遵守浙江省基础公益研究计划相关项目和经费管理规定，并监督实施。

依托单位（公章）：

年 月 日

浙江省自然科学基金委员会办公室审批意见：

同意。

浙江省自然科学基金委员会办公室

2022 年 1 月 10 日

审核通过
