# Supplementary material for: Application of an Exploratory DBT–Based Psychotherapy Program for Suicide‐Related Symptoms in Chinese Outpatients With Affective Disorders
Source: Brain Behav. 2026 Sep 30;16(10):e71827. doi: 10.1002/brb3.71827 (PMC13627894; doi:10.1002/brb3.71827)
Supplement: Supplementary file 2 [file BRB3-16-e71827-s002.docx]

**Contributors**

**Shaohua Hu, Manli Huang, Jianbo Hu:** Conceptialization, Supervision.

**Zhong Wang, Xiaoyi Zhou:** Date curation, Psychotherapy (Methodology).

**Jianbo Lai:** Date curation, Writing-review & editing.

**Yujin Yang:** Writing-original draft.

**Shuguang Li:** Formal analysis, Date curation.

**Jingkai Chen:**Formal analysis.

**Chanchan Hu:** Conceptialization, Writing-review & editing, psychotherapy (Methodology), Funding acquistion.

**Acknowledgements**

I would like to express my gratitude to all those who helped me during the writing of this thesis. I gratefully acknowledge the help of Jianbo Lai, who has offered me valuable suggestions. In the preparation of the thesis, he has spent much time reading through each draft and provided me with inspiring advice. Also thanks to other authors for their help, and joint efforts. I should finally like to express my gratitude to my beloved baby and husband.
